# Randomized Study of Ondansetron Versus Domperidone in the Treatment of Children With Acute Gastroenteritis

**DOI:** 10.4021/jocmr1500w

**Published:** 2013-10-12

**Authors:** Sanguansak Rerksuppaphol, Lakkana Rerksuppaphol

**Affiliations:** a Department of Pediatrics, Faculty of Medicine, Srinakharinwirot University, Nakorn Nayok, Thailand; b Department of Preventive Medicine, Faculty of Medicine, Srinakharinwirot University, Nakorn Nayok, Thailand

**Keywords:** Ondansetron, Domperidone, Acute gastroenteritis, Child

## Abstract

**Background:**

Acute gastroenteritis (AGE) is a common condition among children that is frequently accompanied by vomiting. Symptomatic control of vomiting is important as it improves patient’s general condition and reduces the need for intravenous therapy and hospitalization. Antiemetic agents including ondansetron and domperidone are used to provide symptomatic relief but the existing studies do not provide enough evidence of better efficacy for one over another.

**Methods:**

Seventy-six Thai children under the age of 15 with AGE were randomized to receive either ondansetron or domperidone. The primary outcome of the study was the proportion of the patients in each group who had no episode of vomiting 24 hours after the start of treatment.

**Results:**

Primary outcome was met in 62% of patients in ondansetron group and 44% of patients in domperidone group (P = 0.16). Patients in domperidone group received more doses of the drug within 24 hours after the start of the treatment compared to ondansetron group (P = 0.01). No adverse effect was observed in any of the two groups.

**Conclusions:**

Ondansetron can be considered a safe comparable alternative to commonly-used domperidone in Thai children who suffer from symptoms of gastroenteritis. Larger clinical trials are needed to further explore the effectiveness of the two medications.

## Introduction

Acute gastroenteritis (AGE) is a common cause of morbidity and mortality in children. AGE is frequently accompanied with vomiting that interferes with a successful oral rehydration therapy (ORT). Successful symptomatic management of vomiting not only provides substantial comfort for the patient, but enables the child to be fed orally thus potentially reduces the need for intravenous therapy (IVT) and prolonged hospitalization. Several anti-emetic drugs such as prochlorperazine, promethazine hydrochloride and metoclopramide are used for the management of vomiting; however, their efficacy is limited and comes at the cost of some side effects [1-4]. Currently, there is no standard guideline for pharmacological treatment of vomiting in children with gastroenteritis.

Domperidone is a benzimidazole derivative and a dopamine antagonist that acts on the chemoreceptor trigger zone. It is widely used for the management of vomiting in children [5], however, the evidence of its efficacy is still not satisfactory [6]. A Japanese study of domperidone plus ORT vs. ORT alone in children with AGE showed a trend towards a better control of vomiting within two hours of drug administration in domperidone group, but the difference was not statistically significant [7]. Van Egan et al however, showed that domperidone suppositories significantly decrease the number of vomiting in children with AGE compared to metoclopramide or placebo [8].

Ondansetron is a serotonin (subtype 3) antagonist which has been approved for treatment of nausea and vomiting induced by chemotherapy or radiotherapy as well as for post-operative nausea and vomiting. Compared to dopamine antagonists, it has fewer serious side effects such as extrapyramidal symptoms and demonstrates better efficacy in the treatment of vomiting. This leads to the clinical use of ondansetron off label especially for children’s vomiting caused by AGE [9-11]. Systematic reviews and meta-analyses of the subject concluded that there are few studies on the role of ondansetron in the treatment of vomiting in children. While the evidence is not enough to be conclusive, a trend towards positive efficacy is seen [6, 12, 13] However, there are differences in methodology of studies included in these reviews such as route of administration for medication (orally vs. intravenously) [6]. For example, in a study by Ramsook et al [10] showed that oral ondansetron reduces the episodes of vomiting, the need for IVT, and hospitalization. Similar results were observed by Freedman et al [9] in children treated with a single oral dose of ondansetron in the emergency department. Overall, there is not enough evidence to support the routine use of oral ondansetron in these patients and further studies are needed [14].

Our group has previously reported the efficacy of intravenous ondansetron for the treatment of vomiting in children admitted to hospital [15]. This study will further explore the role of oral ondansetron in an outpatient setting. More specifically, this study aims to determine the efficacy of oral ondansetron compared to domperidone for the treatment of vomiting in children who presented with AGE at the out-patient department of Srinakharinwirot University Hospital in Thailand.

The study is registered into the Thai Clinical Trials Registry with the following trial number: TCTR20120000011.

## Methods

The study was an open-label randomized controlled trial to compare the efficacy of oral disintegrating ondansetron tablet with domperidone suspension in preventing vomiting in children with AGE. The study was conducted in the pediatric out-patient department (OPD) of Srinakharinwirot University Hospital, Thailand between August and December, 2012. The protocol was approved by the Ethic Committee of the Faculty of Medicine, Srinakharinwirot University. Written informed consent and assent were obtained from parents or legal guardians and children, respectively. Children could withdraw from the study at any point during the study.

### Population

Children 15 years of age or younger presenting to the clinic with symptoms consistent with AGE were further assessed for eligibility to participate in the study. The inclusion criteria included: 1) three or more non-bilious, non-bloody vomiting episodes within 24 hours prior to their attendance at clinic, and 2) other signs and symptoms consistent with AGE such as diarrhea, abdominal pain, bloating or discomfort, low grade fever, etc.

Patients who: 1) used any anti-emetic medication within 4 hours prior to the signing of the informed consent form, 2) had underlying diseases such as liver disease, renal disease, congenital heart disease, neurological disease, malignancy, immune deficiency, history of abdominal surgery, and diabetes mellitus, 3) had severe dehydration or severe malnutrition, 4) needed intravenous fluid replacement, 5) had a history of allergy to any anti-emetic medication, or 6) could not tolerate oral feeding were excluded from the study.

### Intervention

Participants were randomized to receive ondansetron or domperidone. Randomization was done using a computerized program with blocks of 2 by a statistician who was not involved in the conduct of the study. Children were given the study treatments at the out-patient clinic. If a child had an immediate episode of vomiting following the administration of the medication, a second dose of the assigned anti-emetic would be administered. Thirty minutes after the administration of anti-emetic, children were allowed to take any other food. Patients were observed at the pediatric outpatient department for one hour after taking the assigned anti-emetic. Those who had no ongoing vomiting were sent home with the assigned anti-emetic and oral dehydration solution (ORS) powder. If the patient still had ongoing vomiting, he/she was re-assessed and was given another dose of the assigned anti-emetic. Children with severe vomiting who could not take any food orally were categorized as treatment failure and were admitted to in-patient unit for proper management (in which case they were taken out of the study). Children who were treated at home were advised to take the assigned anti-emetic at the same dose when they had nausea or ongoing vomiting (PRN; as needed) but only in intervals greater than 8 hours.

#### Ondansetron group

Children received orally disintegrating ondansetron tablet (Zofran Zydis^TM^ GlaxoSmithKline, London, UK) based on their body weight. The prescribed dose was 2 mg for children weighing less than 15 kg, 4 mg for children weighing 15 - 30 kg, and 8 mg for children weighing more than 30 kg.

#### Domperidone group

Children received domperidone suspension (Moridon^®^; New Life Pharma, Bangkok, Thailand) based on their body weight. The prescribed dose was 2.5 mg for children weighing less than 15 kg, 5 mg for children weighing 15 - 30 kg and 10 mg for children weighing more than 30 kg.

All medications were directly purchased from the manufacturing companies. The companies had no role in the conception, design or conduct of the study or in any processes related to the study in any way.

### Data collection and monitoring

Demographic data and clinical data were recorded by nursing staff. Weight was measured to the nearest 100 grams and height was measured to the nearest millimeters. The severity of dehydration was assessed using the Centers for Disease Control and Prevention (CDC) Morbidity and Mortality Weekly Report (MMWR) criteria [16]. Vomiting was defined as any episode of forceful expulsion of stomach content. Two episodes separated by less than 2 minutes were counted as one episode. Treatment, observation and discharge decisions about the patients were done by a pediatrician who was not involved in the implementation phase of the study.

After discharge from the out-patient unit, parents or legal guardians were interviewed via telephone at 24, 48 and 72 hours after treatment by a trained staff to answer some questions about the patients. The questions covered the general condition of patients, number of vomiting episodes, feeding, number of doses of the assigned anti-emetic drug taken, other anti-emetic drugs taken, as well as treatment for this condition from another doctor or hospitalization if applicable. The parents or legal guardians were also asked whether the patient experienced any side effect from the assigned medication, and whether they will be available for the next telephone interview.

### Outcome measurement

The primary outcome was the proportion of children in each group who had no episode of vomiting after 24 hours post treatment. The secondary outcomes were the number of vomiting episodes and percentage of patients who needed further treatment.

### Statistical analysis

To provide the study with a statistical power of 80% and a two-sided type I error of 0.05 to detect 50% vomiting cessation rate in children treated with domperidone and 86% vomiting cessation rate in children treated with ondansetron [9] we needed 31 participants in each arm of the study.

The results were descriptively presented as mean and standard deviation (SD), median and inter-quartile range (IQR), or frequency and percentage. Pearson chi-square or Fisher exact test were used to compare proportions between the groups. The normality of distributions of continuous variables was assessed by Kolmogorov-Sminov test. Not-normally distributed continuous data were compared using Mann-Whitney U test whereas normally distributed data were compared by a student t-test. Times to event (cessation of vomiting) were evaluated by the Kaplan-Meier survival analysis and compared by the log-rank test. Statistical analysis was performed with SPSS 16.0 software (SPSS. Inc, Chicago, IL). P-value of less than 0.05 was considered significant.

## Results

**Figure 1:**
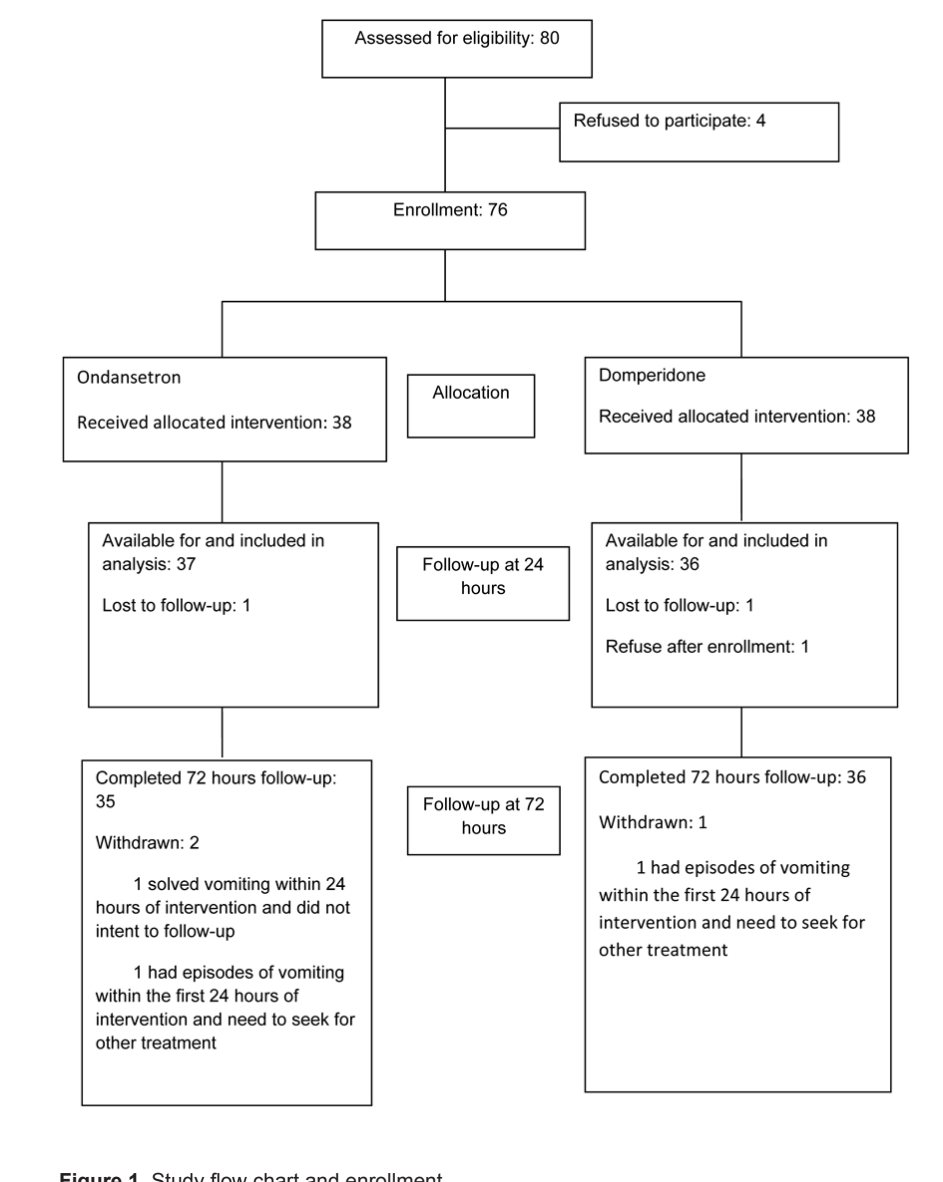
Study flow chart and enrollment.

Eighty previously healthy children diagnosed with AGE were approached to participate in the study. Seventy-six children, median age 3.8 years (range 0.4 - 14.6 years), whose parents consented entered the study and were randomized to receive either ondansetron (n = 38) or domperidone (n = 38). A study flow chart and enrollment is reported in Figure 1.

One child in domperidone group was excluded due to the fact that his parents withdrew their consent shortly after enrolment. In addition, parents of one child in each group could not be reached by phone at 24 hours follow-up and these two children were excluded from the analysis. Therefore, a total of 73 children, 37 in ondansetron and 36 in domperidone group, were included in the primary analysis at 24 hours follow-up. Two children in ondansetron group withdrew from the study at 48 hours follow-up. One of them stopped vomiting after the administration of the first dose at the out-patient clinic and did not receive a phone call due to travelling. Another child had 4 vomits in the first 24 hours of the study and sought further treatment by visiting a doctor at another hospital. One child in domperidone group had withdrawn from the study at 48 hours follow-up because of ongoing vomiting and sought other treatments. Eventually, this patient received intravenous metoclopramide and was admitted to hospital. In total, seventy children were available for follow-up at 48 and 72 hours and were included in the follow-up analysis.

Baseline characteristics of participants were comparable between two groups as shown in Table 1. The majority of the study population (93%) had no or mild dehydration at the time of enrolment. At the beginning of the study, all children were able to take the assigned anti-emetics at the OPD and there were no immediate vomiting reported after the start of medications. All children had been discharged to stay at home without any need for a repeat dose during the one hour observation period at OPD.

**Table 1:** Baseline Characteristics of the Participants

Characteristics	Ondansetron (n = 38)	Domperidone (n = 38)	P-value
Age (yr); mean (SD)	3.7 (0.5)	4.7 (0.5)	0.115
Boy; n (%)	20 (52.6)	21 (55.3)	1.000
Weight (kg)	16.7 (1.7)	17.8 (1.3)	0.169
Height (cm); mean (SD)	99.1 (3.6)	106.8 (2.9)	0.169
Duration of vomiting before enrolment (hours); Median (IQR)	12.0 (6.8 - 30.0)	12.0 (5.0 - 24.0)	0.634
Number of vomiting episodes in preceding 24 hours; Median (IQR)	4.5 (3.0 - 6.3)	3.0 (3.0 - 5.3)	0.136
Time of last vomiting before enrolment (hours); Median (IQR)	3.0 (1.0 - 5.0)	2.0 (1.4 - 4.0)	0.962
Hydration status; n (%)			0.358
Minimal or no dehydration	34 (89.5)	37 (97.4)	
Moderate	4 (10.5)	1 (2.6)	
Presence of diarrhea; n (%)	16 (42.1)	18 (47.4)	0.818
Presence of fever; n (%)	25 (65.8)	25 (65.8)	1.000

By 24-hour follow-up, the vomiting stopped in 62% (23/37) of patients in ondansetron group and in 44% (16/36) of patients in domperidone group (P = 0.16). Median number of vomiting episodes post treatment was comparable between the groups as shown in Table 2 (0 in ondansetron vs. 1 in domperidone; P = 0.085).

**Table 2:** Outcome Measures - Ondansetron Versus Domperidone Group

	Ondansetron	Domperidone	P-value
24 hours after treatment			
Vomiting; n (%)	14 (37.8)	20 (55.6)	0.162
Vomiting episode; Median (IQR)	0 (0 - 1)	1 (0 - 2)	0.085
Vomiting episodes per patient; n (%)			0.148
0	23 (62.2)	16 (44.4)	
1	10 (27.0)	10 (27.8)	
2	4 (10.8)	10 (27.8)	
Number of children took additional dose of the assigned anti-emetic; n (%)	8/14 (57.1)	17/20 (85.0)	0.116
Number of doses taken; median (IQR)	1 (0 - 1)	1.5 (1 - 2)	0.011
Vomiting at 24-48 hrs; n (%)	2 (5.7)	4 (11.4)	0.673
Vomiting at 48-72 hrs; n (%)	1 (2.9)	2 (5.7)	1.000

Of 14 children in ondansetron group who continued to have vomiting beyond 24 hours post treatment, 8 (57.1%) took at least one additional dose compared to 17 of 20 (85%) children in domperidone group (P = 0.11). In addition, patients in ondansetron group - on average - took a lower number of additional doses of the assigned anti-emetic drug overall compared to domperidone group (median: 1 vs. 1.5 doses, respectively; P = 0.01).

Two children (5.7%) in ondansetron group had vomiting at the second follow-up (48 hours) compared to 4 (11.4%) children in domperidone group (P = 0.673). One child in each group took additional dose of the assigned anti-emetic without a need for further treatment. At the third day follow-up point, one child in ondansetron arm and 2 children in domperidone arm had vomiting, however, only 1 child in domperidone group took an additional dose. Children had good conditions and did not need further treatment or hospital admission.

**Figure 2:**
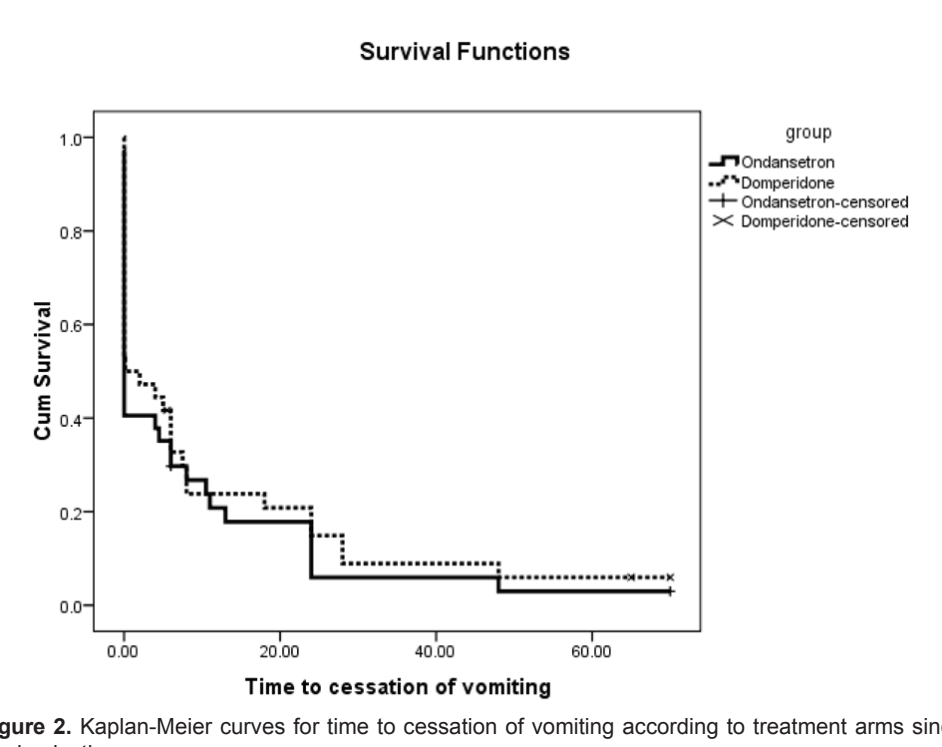
Kaplan-Meier curves for time to cessation of vomiting according to treatment arms since randomization.

Kaplan-Meier survival analysis indicated that the estimated time to cessation of vomiting in ondansetron group was 8.2 hours (95%CI: 3.2 - 13.2 hours) and was comparable with domperidone group at 10.8 hours (95%CI: 4.6 - 17.0 hours) (P = 0.485). Kaplan-Meier curves for children treated with ondansetron and domperidone are shown in Figure 2.

With regards to safety, there were no reports of adverse effects during the study in neither group.

## Discussion

Although a trend towards better efficacy for ondansetron was seen, the study could not demonstrate a statistically significant difference between ondansetron and domperidone in controlling vomiting of patients with AGE. However, patients in domperidone group on average had to receive a higher number of study medication compared to the patients in ondansetron group.

The choice of domperidone for this study was primarily because it is commonly used in this population in Thailand. Although the efficacy of domperidone is not fully established, we considered it the standard of care and compared ondansetron against it. There were also discussions of using placebo as the control group but because of ethical reasons we preferred the use of an active control over placebo.

It can be hypothesized that one reason for the lack of significant difference with regards to our main study outcome is the fact that we only included patients with mild to moderate disease (mainly mild disease) and excluded those with severe dehydration. Mild/moderate disease is known to have a benign trajectory and is often self-limiting. This can potentially dilute the positive effect of anti-emetics and make it harder to differentiate a superior treatment.

The cessation rates of vomiting in our study appear to be less than most of studies in children that used ondansetron [9, 10, 17, 18]. We think that the main reason for the difference is the fact that each study uses a different dosage or route of administration of the drug, is done in a slightly different population, evaluates vomiting at different timepoint, and/or has a different methodology. In terms of safety, our findings are consistent with other similar studies; both drugs were generally well tolerated by patients and no adverse effect was reported.

The use of different forms of ondansetron in children with nausea and vomiting has been the subject of many studies in the past. Orally disintegrating ondansetron tablet in particular, provides a convenient route of administration and is easier than regular tablets to be administered to children with vomiting. It is also less invasive than IV ondansetron especially in those children who are planned to be treated as an outpatient [19]. Our study also demonstrates that orally disintegrating ondansetron is well-accepted among children who suffer from symptoms of AGE.

The need for ORS could potentially be considered an interesting secondary outcome for this study. However, we did not evaluate that for feasibility reasons. We thought that simply asking the parents over the phone the question of whether the child received ORS would not be adequately reliable.

Lack of blindness is a potential limitation of this study. The medications are different in shape and patients as well as doctors were aware of the name of the study medication the patient received. Because of the medications were in different forms (tablet vs. suspension), it was difficult to ensure the blindness of the nursing staff who made the phone calls and collected the information at follow-up and as a result most of them could identify the patient’s arm at the end of their conversation with parents.

### Conclusion

Our study suggests that both ondansetron and domperidone can be used in treating children suffering from symptoms of AGE. They both demonstrate an acceptable efficacy as well as a good safety profile. Most Children who can tolerate the first dose can be safely sent home after essential instructions for parents are provided. Most of the patients will recover from their symptoms within 72 hours after the start of treatment. Further studies including a large multi-center randomized controlled trial [20] that is currently being implemented will hopefully provide answers to some of the specific questions regarding the possible role of ondansetron in children with AGE.

## References

[1] DeGrandi T, Simon JE (1987). Promethazine-induced dystonic reaction. Pediatr Emerg Care.

[2] Leary PM (1991). Adverse reactions in children. Special considerations in prevention and management. Drug Saf.

[3] Bateman DN, Darling WM, Boys R, Rawlins MD (1989). Extrapyramidal reactions to metoclopramide and prochlorperazine. Q J Med.

[4] Boulloche J, Mallet E, Mouterde O, Tron P (1987). Dystonic reactions with metoclopramide: is there a risk population?. Helv Paediatr Acta.

[5] Albano F, Bruzzese E, Spagnuolo MI, De Marco G (2006). Antiemetics for children with gastroenteritis: off-label but still on in clinical practice. J Pediatr Gastroenterol Nutr.

[6] DeCamp LR, Byerley JS, Doshi N, Steiner MJ (2008). Use of antiemetic agents in acute gastroenteritis: a systematic review and meta-analysis. Arch Pediatr Adolesc Med.

[7] Kita F, Hinotsu S, Yorifuji T, Urushihara H, Shimakawa T, Kishida K, Wakazono Y (2012). Domperidone With ORT in the Treatment of Pediatric Acute Gastroenteritis in Japan: A Multicenter, Randomized Controlled Trial. Asia Pac J Public Health.

[8] Van Eygen M, Dhondt F, Heck E, Ameryckx L, Van Ravensteyn H (1979). A double-blind comparison of domperidone and metoclopramide suppositories in the treatment of nausea and vomiting in children. Postgrad Med J.

[9] Freedman SB, Adler M, Seshadri R, Powell EC (2006). Oral ondansetron for gastroenteritis in a pediatric emergency department. N Engl J Med.

[10] Ramsook C, Sahagun-Carreon I, Kozinetz CA, Moro-Sutherland D (2002). A randomized clinical trial comparing oral ondansetron with placebo in children with vomiting from acute gastroenteritis. Ann Emerg Med.

[11] Reeves JJ, Shannon MW, Fleisher GR (2002). Ondansetron decreases vomiting associated with acute gastroenteritis: a randomized, controlled trial. Pediatrics.

[12] Alhashimi D, Al-Hashimi H, Fedorowicz Z (2009). Antiemetics for reducing vomiting related to acute gastroenteritis in children and adolescents. Cochrane Database Syst Rev.

[13] Szajewska H, Gieruszczak-Bialek D, Dylag M (2007). Meta-analysis: ondansetron for vomiting in acute gastroenteritis in children. Aliment Pharmacol Ther.

[14] Borowitz SM (2005). Are antiemetics helpful in young children suffering from acute viral gastroenteritis?. Arch Dis Child.

[15] Rerksuppaphol S, Rerksuppaphol L (2010). Efficacy of intravenous ondansetron to prevent vomiting episodes in acute gastroenteritis: a randomized, double blind, and controlled trial. Pediatr Rep.

[16] King CK, Glass R, Bresee JS, Duggan C (2003). Managing acute gastroenteritis among children: oral rehydration, maintenance, and nutritional therapy. MMWR Recomm Rep.

[17] Roslund G, Hepps TS, McQuillen KK (2008). The role of oral ondansetron in children with vomiting as a result of acute gastritis/gastroenteritis who have failed oral rehydration therapy: a randomized controlled trial. Ann Emerg Med.

[18] Yilmaz HL, Yildizdas RD, Sertdemir Y (2010). Clinical trial: oral ondansetron for reducing vomiting secondary to acute gastroenteritis in children--a double-blind randomized study. Aliment Pharmacol Ther.

[19] Cohen IT, Joffe D, Hummer K, Soluri A (2005). Ondansetron oral disintegrating tablets: acceptability and efficacy in children undergoing adenotonsillectomy. Anesth Analg.

[20] Marchetti F, Maestro A, Rovere F, Zanon D, Arrighini A, Bertolani P, Biban P (2011). Oral ondansetron versus domperidone for symptomatic treatment of vomiting during acute gastroenteritis in children: multicentre randomized controlled trial. BMC Pediatr.

